# 1*H*-Benzo[*g*]pteridine-2,4-dione

**DOI:** 10.1107/S2414314622012238

**Published:** 2023-01-06

**Authors:** Rao M. Uppu, Frank R. Fronczek

**Affiliations:** a Department of Environmental Toxicology, Southern University and A&M College, Baton Rouge, LA 70813, USA; b Department of Chemistry, Louisiana State University, Baton Rouge, LA 70803, USA; University of Aberdeen, United Kingdom

**Keywords:** alloxazine, Isoalloxazine, crystal structure, tautomerism, hydrogen bonding

## Abstract

The structure of the title compound, C_10_H_6_N_4_O_2_, reported by Smalley *et al.* [(2021). *Cryst. Growth Des.* **22**, 524–534] from powder diffraction data and ^15^N NMR spectroscopy, is confirmed using low-temperature data from a twinned crystal. The tautomer in the solid state is alloxazine (1*H*-benzo[*g*]pteridine-2,4-dione) rather than isoalloxazine (10*H*-benzo[*g*]pteridine-2,4-dione). In the extended structure, the mol­ecules form hydrogen-bonded chains propagating in the [0  1] direction through alternating centrosymmetric *R* ^2^_2_(8) rings with pairwise N—H⋯O inter­actions and centrosymmetric *R* ^2^_2_(8) rings with pairwise N—H⋯N inter­actions. The crystal chosen for data collection was found to be a non-merohedral twin (180° rotation about [001]) in a 0.446 (4):0.554 (6) domain ratio.

## Abstract 2

The structure of the title compound, reported from powder diffraction data and ^15^N NMR spectroscopy, is confirmed using low-temperature data from a twinned crystal. The tautomer in the solid state is alloxazine (1*H*-benzo[*g*]pteridine-2,4-dione) rather than isoalloxazine (10*H*-benzo[*g*]pteridine-2,4-dione).

## Graphical abstract

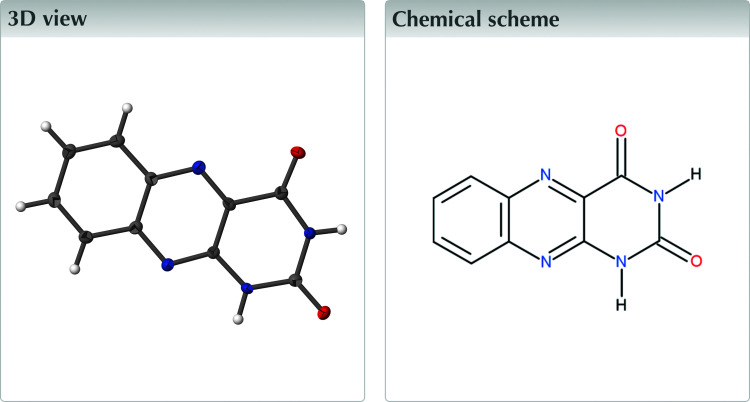

## Structure description

1*H*-Benzo[*g*]pteridine-2,4-dione, popularly known as alloxazine, is a tautomer of isoalloxazine (10*H*-benzo[*g*]pteridine-2,4-dione), the same ring system that is present in riboflavin, flavin nucleotides (FMN and FAD), and flavoproteins. Unlike nicotinamide coenzymes, NAD(P)^+^ and NAD(P)H, flavin nucleotides serve in both one-electron and two-electron transfer reactions because the isoalloxazine ring can exist in several different ionization and/or redox states (Massey & Hemmerich, 1980 ▸). Further, the strong but mostly non-covalent inter­actions within the flavoprotein binding site allow the fine-tuning of the redox chemistry of the isoalloxazine ring system (Ghisla *et al.*, 1974 ▸; Hu *et al.*, 2015 ▸; van den Heuvel *et al.*, 2002 ▸) which, among many things, helps in minimizing the 1-electron reduction of mol­ecular oxygen to the superoxide anion radical. It is believed that the spatial arrangement of the reacting oxygen mol­ecule may have a direct bearing on the outcome of a flavoprotein serving as an oxidase or de­hydrogenase function (Chaiyen *et al.*, 2012 ▸), a process that can be mimicked in simple chemical model systems of phenazine reacting with NAD(P)H in micelle forming surfactant solutions (Nishikimi *et al.*, 1972 ▸; Rao, 1989*a* ▸,*b* ▸; Uppu, 1995 ▸). While there have been several efforts to define flavin–protein inter­actions that have mainly capitalized on differences in the chemical reactivity of the protein-bound flavin, we were surprised to note that, except for one recent study by Smalley *et al.* (2022 ▸), there are hardly any studies of the crystal structure of alloxazine itself.

**Figure 1:**
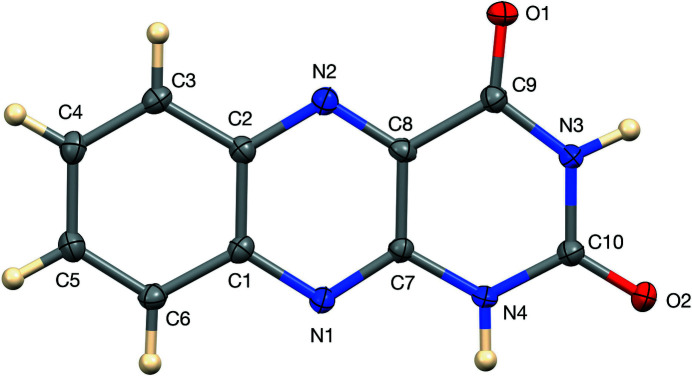
The title mol­ecule with 50% ellipsoids.

In view of the above and since two-thirds of flavoprotein allelic variants are linked to human diseases (Lienhart *et al.* 2013 ▸), we determined the crystal structure of alloxazine using a Bruker Kappa APEXII DUO diffractometer. Using low-temperature (90 K) data from twinned crystals, our results confirm the observations of Smalley *et al.* (2022 ▸), who used powder diffraction data along with ^15^N NMR spectroscopy. The tautomer in the solid state is alloxazine rather than isoalloxazine. The N-bound hydrogen atoms were located and their positions were refined in order to confirm the tautomer. The mol­ecule, shown in Fig. 1 ▸ is nearly planar, with an r.m.s deviation for 16 non-hydrogen atoms of 0.015 Å and a maximum deviation of 0.025 (3) Å for C5.

**Figure 2:**
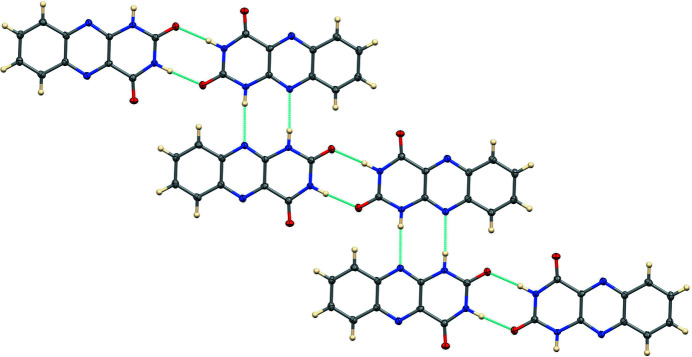
The hydrogen-bonded chain.

**Figure 3:**
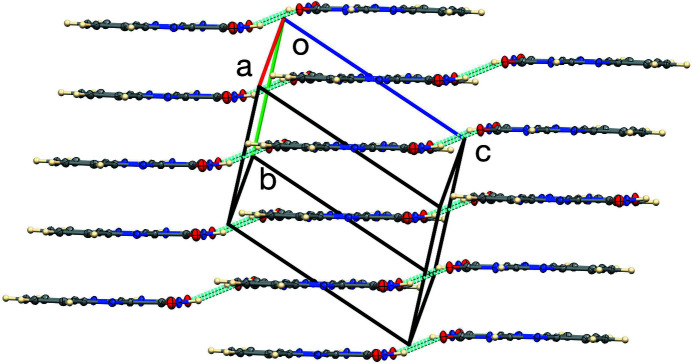
The unit cell, showing the offsets of hydrogen-bonded dimers.

The inter­molecular hydrogen bonding (Table 1 ▸) is shown in Fig. 2 ▸. Atom N4 donates a hydrogen bond to N1, forming an 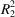 (8) ring (Etter *et al.*, 1990 ▸) about the inversion center at 0,1,½. Similarly, N3 donates a hydrogen bond to O2, forming a centrosymmetric 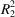 (8) ring about 0,½,1. Thus, these two pairs of inter­actions combine to form a hydrogen-bonded chain propagating in the [0 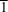 1] direction. The planes of the N,N dimers related by the N—H⋯O hydrogen bonds are offset by 0.915 (2) Å, as illus­trated in Fig. 3 ▸.

## Synthesis and crystallization

The title compound, C_10_H_6_N_4_O_2_ (alloxazine) was obtained from Sigma-Aldrich, St. Louis, Missouri, USA and was used without further purification. Single crystals in the form of pale yellow plates were prepared by slow cooling of a nearly saturated solution of alloxazine in dimethyl formamide at 135 ± 2°C

## Refinement

Crystal data, data collection and structure refinement details are summarized in Table 2 ▸. The crystal chosen for data collection was refined as a two-component non-merohedral twin, by 180° rotation about the reciprocal [001] direction. Both twin components were integrated. Refinement was against an HKLF 5 file prepared using *TWINABS*. The refined BASF parameter is 0.446 (4). Seven outlier reflections were omitted from the refinement.

## Supplementary Material

Crystal structure: contains datablock(s) I. DOI: 10.1107/S2414314622012238/hb4421sup1.cif

Structure factors: contains datablock(s) I. DOI: 10.1107/S2414314622012238/hb4421Isup2.hkl

Supporting information file. DOI: 10.1107/S2414314622012238/hb4421Isup3.cml

CCDC reference: 2233313

Additional supporting information:  crystallographic information; 3D view; checkCIF report

## Figures and Tables

**Table 1:** Hydrogen-bond geometry (Å, °)

*D*—H⋯*A*	*D*—H	H⋯*A*	*D*⋯*A*	*D*—H⋯*A*
N3—H3*N*⋯O2^i^	0.92 (3)	2.00 (4)	2.883 (3)	162 (3)
N4—H4*N*⋯N1^ii^	0.90 (3)	2.22 (3)	3.114 (3)	176 (3)
C3—H3⋯N2^iii^	0.95	2.58	3.520 (4)	173
C6—H6⋯O2^ii^	0.95	2.21	3.158 (4)	173

Symmetry codes: (i) 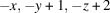 ; (ii) 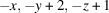 ; (iii) 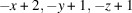 .

**Table 2:** Experimental details

Crystal data	
Chemical formula	C_10_H_6_N_4_O_2_
*M*_r_	214.19
Crystal system, space group	Triclinic, *P* 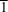
Temperature (K)	90
*a*, *b*, *c* (Å)	5.8027 (2), 7.5404 (3), 10.1345 (4)
α, β, γ (°)	70.483 (2), 84.150 (2), 84.208 (3)
*V* (Å^3^)	414.72 (3)
*Z*	2
Radiation type	Cu *K*α
μ (mm^−1^)	1.06
Crystal size (mm)	0.11 × 0.06 × 0.02

Data collection	
Diffractometer	Bruker Kappa APEXII DUO CCD
Absorption correction	Multi-scan (*TWINABS*; Bruker, 2001 ▸)
*T*_min_, *T*_max_	0.774, 0.979
No. of measured, independent and observed [*I* > 2σ(*I*)] reflections	6802, 6802, 4832
*R*_int_	?
(sin θ/λ)_max_ (Å^−1^)	0.607

Refinement	
*R*[*F* ^2^ > 2σ(*F* ^2^)], *wR*(*F* ^2^), *S*	0.048, 0.144, 1.07
No. of reflections	6802
No. of parameters	152
H-atom treatment	H atoms treated by a mixture of independent and constrained refinement
Δρ_max_, Δρ_min_ (e Å^−3^)	0.26, −0.28

Computer programs: *APEX2* (Bruker, 2016 ▸), *SAINT* (Bruker, 2016 ▸), *SHELXT2014/5* (Sheldrick, 2015*a* ▸), *SHELXL2017/1* (Sheldrick, 2015*b* ▸), *Mercury* (Macrae *et al.*, 2020 ▸), and *publCIF* (Westrip, 2010 ▸).

## full crystallographic data

### Crystal data

**Table:**

C_10_H_6_N_4_O_2_	*Z* = 2
*M**_r_* = 214.19	*F*(000) = 220
Triclinic, *P*1	*D*_x_ = 1.715 Mg m^−^^3^
*a* = 5.8027 (2) Å	Cu *K*α radiation, λ = 1.54184 Å
*b* = 7.5404 (3) Å	Cell parameters from 4433 reflections
*c* = 10.1345 (4) Å	θ = 4.6–69.1°
α = 70.483 (2)°	µ = 1.06 mm^−^^1^
β = 84.150 (2)°	*T* = 90 K
γ = 84.208 (3)°	Plate, pale yellow
*V* = 414.72 (3) Å^3^	0.11 × 0.06 × 0.02 mm

### Data collection

**Table:**

Bruker Kappa APEXII DUO CCD diffractometer	6802 independent reflections
Radiation source: IµS microfocus	4832 reflections with *I* > 2σ(*I*)
QUAZAR multilayer optics monochromator	θ_max_ = 69.4°, θ_min_ = 4.6°
φ and ω scans	*h* = −7→7
Absorption correction: multi-scan (*TWINABS*; Bruker, 2001)	*k* = −9→9
*T*_min_ = 0.774, *T*_max_ = 0.979	*l* = −12→12
6802 measured reflections	

### Refinement

**Table:**

Refinement on *F*^2^	Primary atom site location: dual
Least-squares matrix: full	Hydrogen site location: mixed
*R*[*F*^2^ > 2σ(*F*^2^)] = 0.048	H atoms treated by a mixture of independent and constrained refinement
*wR*(*F*^2^) = 0.144	*w* = 1/[σ^2^(*F*_o_^2^) + (0.0713*P*)^2^ + 0.0047*P*] where *P* = (*F*_o_^2^ + 2*F*_c_^2^)/3
*S* = 1.07	(Δ/σ)_max_ < 0.001
6802 reflections	Δρ_max_ = 0.26 e Å^−^^3^
152 parameters	Δρ_min_ = −0.28 e Å^−^^3^
0 restraints	

### Special details

**Table:**

Geometry. All esds (except the esd in the dihedral angle between two l.s. planes) are estimated using the full covariance matrix. The cell esds are taken into account individually in the estimation of esds in distances, angles and torsion angles; correlations between esds in cell parameters are only used when they are defined by crystal symmetry. An approximate (isotropic) treatment of cell esds is used for estimating esds involving l.s. planes.
Refinement. Refined as a 2-component twin, by 180 deg. rotation about reciprocal 0 0 1. Refinement was vs. an HKLF 5 file prepared using TWINABS. The refined BASF parameter is 0.446 (4). Seven outlier reflections were omitted from the refinement.All H atoms were located in difference maps and those on C were thereafter treated as riding in geometrically idealized positions with C—H distances 0.95 Å. Coordinates of the N—H hydrogen atom were refined. *U*_iso_(H) values were assigned as 1.2*U*_eq_ of the attached atom.

### Fractional atomic coordinates and isotropic or equivalent isotropic displacement parameters (Å^2^)

**Table:**

	*x*	*y*	*z*	*U*_iso_*/*U*_eq_	
O1	0.6141 (4)	0.4107 (3)	0.8404 (2)	0.0215 (6)	
O2	−0.0946 (3)	0.7091 (3)	0.8834 (2)	0.0213 (6)	
N1	0.2942 (4)	0.8937 (4)	0.4485 (2)	0.0150 (6)	
N2	0.6687 (4)	0.6283 (4)	0.5564 (2)	0.0160 (6)	
N3	0.2614 (4)	0.5646 (4)	0.8587 (3)	0.0166 (6)	
H3N	0.231 (5)	0.487 (5)	0.949 (4)	0.020*	
N4	0.1014 (4)	0.7992 (4)	0.6692 (2)	0.0160 (6)	
H4N	−0.018 (6)	0.884 (5)	0.638 (3)	0.019*	
C1	0.4878 (5)	0.8700 (4)	0.3647 (3)	0.0154 (7)	
C2	0.6759 (5)	0.7382 (4)	0.4193 (3)	0.0154 (7)	
C3	0.8747 (5)	0.7183 (4)	0.3300 (3)	0.0165 (7)	
H3	1.001181	0.631113	0.366387	0.020*	
C4	0.8827 (5)	0.8255 (5)	0.1911 (3)	0.0181 (7)	
H4	1.014200	0.811737	0.130392	0.022*	
C5	0.6963 (5)	0.9562 (5)	0.1379 (3)	0.0179 (7)	
H5	0.704994	1.030108	0.041346	0.022*	
C6	0.5034 (5)	0.9801 (5)	0.2209 (3)	0.0171 (7)	
H6	0.380553	1.069981	0.182484	0.020*	
C7	0.2926 (5)	0.7827 (4)	0.5806 (3)	0.0135 (6)	
C8	0.4803 (5)	0.6503 (4)	0.6354 (3)	0.0147 (7)	
C9	0.4648 (5)	0.5301 (5)	0.7850 (3)	0.0163 (7)	
C10	0.0784 (5)	0.6913 (5)	0.8079 (3)	0.0164 (7)	

### Atomic displacement parameters (Å^2^)

**Table:**

	*U*^11^	*U*^22^	*U*^33^	*U*^12^	*U*^13^	*U*^23^
O1	0.0173 (11)	0.0228 (13)	0.0192 (11)	0.0062 (9)	−0.0022 (9)	−0.0018 (9)
O2	0.0187 (11)	0.0237 (13)	0.0164 (10)	0.0027 (9)	0.0043 (9)	−0.0024 (9)
N1	0.0137 (12)	0.0175 (15)	0.0133 (13)	−0.0003 (10)	−0.0001 (10)	−0.0047 (11)
N2	0.0168 (12)	0.0151 (14)	0.0160 (13)	−0.0017 (10)	0.0009 (10)	−0.0055 (11)
N3	0.0150 (13)	0.0179 (14)	0.0124 (12)	0.0033 (10)	0.0007 (10)	−0.0008 (10)
N4	0.0141 (13)	0.0180 (14)	0.0125 (12)	0.0046 (11)	−0.0020 (10)	−0.0016 (10)
C1	0.0145 (14)	0.0168 (16)	0.0163 (16)	−0.0015 (12)	−0.0001 (12)	−0.0074 (13)
C2	0.0159 (14)	0.0156 (16)	0.0143 (15)	−0.0015 (12)	−0.0017 (12)	−0.0040 (13)
C3	0.0139 (14)	0.0174 (17)	0.0199 (15)	−0.0003 (12)	−0.0012 (12)	−0.0085 (13)
C4	0.0164 (15)	0.0220 (17)	0.0167 (15)	−0.0043 (12)	0.0015 (12)	−0.0071 (12)
C5	0.0197 (15)	0.0195 (17)	0.0140 (15)	−0.0025 (12)	−0.0010 (12)	−0.0044 (12)
C6	0.0151 (14)	0.0195 (17)	0.0170 (15)	0.0018 (12)	−0.0030 (12)	−0.0068 (13)
C7	0.0140 (14)	0.0127 (16)	0.0139 (14)	−0.0014 (11)	0.0000 (12)	−0.0048 (12)
C8	0.0159 (15)	0.0149 (16)	0.0133 (16)	0.0006 (12)	−0.0048 (12)	−0.0039 (13)
C9	0.0162 (15)	0.0176 (17)	0.0152 (16)	−0.0007 (12)	−0.0014 (12)	−0.0056 (13)
C10	0.0155 (15)	0.0176 (17)	0.0158 (15)	0.0010 (12)	−0.0013 (12)	−0.0056 (12)

### Geometric parameters (Å, º)

**Table:**

O1—C9	1.217 (3)	C1—C6	1.414 (4)
O2—C10	1.224 (4)	C1—C2	1.422 (4)
N1—C7	1.321 (4)	C2—C3	1.420 (4)
N1—C1	1.372 (4)	C3—C4	1.368 (4)
N2—C8	1.318 (4)	C3—H3	0.9500
N2—C2	1.358 (4)	C4—C5	1.407 (4)
N3—C10	1.372 (4)	C4—H4	0.9500
N3—C9	1.381 (4)	C5—C6	1.364 (4)
N3—H3N	0.92 (3)	C5—H5	0.9500
N4—C10	1.369 (4)	C6—H6	0.9500
N4—C7	1.377 (4)	C7—C8	1.423 (4)
N4—H4N	0.90 (3)	C8—C9	1.483 (4)
			
C7—N1—C1	115.5 (3)	C5—C4—H4	120.0
C8—N2—C2	116.9 (3)	C6—C5—C4	122.0 (3)
C10—N3—C9	127.3 (3)	C6—C5—H5	119.0
C10—N3—H3N	113 (2)	C4—C5—H5	119.0
C9—N3—H3N	119 (2)	C5—C6—C1	119.5 (3)
C10—N4—C7	123.5 (3)	C5—C6—H6	120.2
C10—N4—H4N	116 (2)	C1—C6—H6	120.2
C7—N4—H4N	121 (2)	N1—C7—N4	118.0 (3)
N1—C1—C6	119.7 (3)	N1—C7—C8	123.0 (3)
N1—C1—C2	121.5 (3)	N4—C7—C8	119.0 (3)
C6—C1—C2	118.8 (3)	N2—C8—C7	122.0 (3)
N2—C2—C3	118.8 (3)	N2—C8—C9	118.3 (3)
N2—C2—C1	121.1 (3)	C7—C8—C9	119.8 (3)
C3—C2—C1	120.1 (3)	O1—C9—N3	121.7 (3)
C4—C3—C2	119.5 (3)	O1—C9—C8	124.5 (3)
C4—C3—H3	120.3	N3—C9—C8	113.8 (3)
C2—C3—H3	120.3	O2—C10—N4	121.8 (3)
C3—C4—C5	120.1 (3)	O2—C10—N3	121.6 (3)
C3—C4—H4	120.0	N4—C10—N3	116.6 (3)

### Hydrogen-bond geometry (Å, º)

**Table:**

*D*—H···*A*	*D*—H	H···*A*	*D*···*A*	*D*—H···*A*
N3—H3*N*···O2^i^	0.92 (3)	2.00 (4)	2.883 (3)	162 (3)
N4—H4*N*···N1^ii^	0.90 (3)	2.22 (3)	3.114 (3)	176 (3)
C3—H3···N2^iii^	0.95	2.58	3.520 (4)	173
C6—H6···O2^ii^	0.95	2.21	3.158 (4)	173

Symmetry codes: (i) −*x*, −*y*+1, −*z*+2; (ii) −*x*, −*y*+2, −*z*+1; (iii) −*x*+2, −*y*+1, −*z*+1.
